# Genome expression analysis of basic helix-loop-helix transcription factors in Sea buckthorn (*Hippophae rhamnoides* L.)

**DOI:** 10.3389/fpls.2024.1487960

**Published:** 2024-11-20

**Authors:** Jiajia Zhang, Gaigai Du, Guoyun Zhang, Jianguo Zhang, Songfeng Diao

**Affiliations:** ^1^ Key Laboratory of Cultivation and Protection for Non-Wood Forest Trees of the Ministry of Education, Central South University of Forestry and Technology, Changsha, China; ^2^ Research Institute of Non-timber Forestry, Chinese Academy of Forestry, Key Laboratory of Non-timber Forest Germplasm Enhancement and Utilization of National Forestry and Grassland Administration, Zhengzhou, China; ^3^ Radboud Universitieit, Nijmegen, Netherlands; ^4^ Research Institute of Forestry, Chinese Academy of Forestry, State Key Laboratory of Tree Genetics and Breeding, Beijing, China

**Keywords:** Hippophae rhamnoides, genome-wide analysis, basic helix-loop-helix TFs, expression analysis, fruit development

## Abstract

**Introduction:**

The basic helix-loop-helix (bHLH) transcription factor family is one of the largest gene families in plants, extensively involved in plant growth, organ development, and stress responses. However, limited studies of this family are available in sea buckthorn (*Hippophae rhamnoides*).

**Methods:**

In this study, we identified 144 bHLH genes in *H. rhamnoides* (HrbHLH) through a genome-wide search method, then explored their DNA and protein sequences and physicochemical properties.

**Results and discussion:**

According to the sequence similarities, we classified them into 15 groups with specific motif structures. To explore their expressions, we performed gene expression profiling using RNA-Seq and identified 122 HrbHLH mRNAs were highly expressed, while the remaining 22 HrbHLH genes were expressed at very low levels in all 21 samples. Among these HrbHLH genes, *HrbHLH47, HrbHLH74, HrbHLH90, HrbHLH131* showed the highest expression level in the root nodule, root, leaf, stem and fruit tissues. Furthermore, eleven HrbHLH genes displayed increased expressions during the fruit development process of sea buckthorn. Finally, we validated the expression patterns of HrbHLH genes using reverse transcription quantitative real-time PCR (QPCR). This comprehensive analysis provides a useful esource that enables further investigation of the physiological roles and molecular functions of the HrbHLH TFs.

## Introduction

The basic helix-loop-helix (bHLH) transcription factors family widely exists in eukaryotes and plays an important role in plant growth, organ development and stress response (Carretero-Paulet et al., 2010). Until now, more than 100 bHLH proteins have been found and functionally analyzed in animals and plants since the bHLH protein structure was firstly analyzed (Ludwig and Wessler, 1990). The bHLH gene family is named for its highly conserved bHLH domain, which consists of about 60 amino acids, including the basic region distributed at the N-terminus and the helix-loop-helix (HLH) region distributed at the C-terminus of the polypeptide chain (Heim et al., 2003). In the basic region, 15 amino acid residues are involved in DNA recognition and binding. The HLH region includes two amphiphilic α-helices and a loop structure, which can form homodimer or heterodimeric with other proteins. According to previous studies, animal bHLH proteins can be divided into six groups (A-F). In plants, bHLH proteins are categorized into more groups than the six typically seen in animals. Based on studies, plant bHLH proteins are divided into 12-15 groups, depending on the classification system used. Some groups are unique to plants that include, group B, C, D, E, F and K (Pires and Dolan, 2010). However, many identified bHLH proteins are involved in group B in plants, characterized by binding to G-box (Wang et al., 2018). The basic helix-loop-helix (bHLH) transcription factors have been demonstrated to have diverse functions in plant growth, development and stress responses. For example, the bHLH genes SPATULA and ALCATRAZ affect carpel and fruit development in *Arabidopsis* (Groszmann et al., 2011, 2008). And, three bHLH genes (MYC2, MYC3, and MYC4) can affect the flowering of *Arabidopsis thaliana* (Wang et al., 2017). The bHLH gene BIGPETALp in A. thaliana is important in controlling petal size (Szecsi et al., 2006). Past research showed that the bHLH gene BIM1 is associated with embryonic patterning (Chandler et al., 2009). MdbHLH3 gene promotes anthocyanin accumulation and fruit coloration in apple (Xie et al., 2012). SlPRE2 affects plant morphology and fruit pigment accumulation in tomato (Zhu et al., 2017). The bHLH genes also regulate plant response to various abiotic stresses, such as drought (Li et al., 2019; Waseem et al., 2019; Yao et al., 2017), salinity (Chen et al., 2018; Samira et al., 2018; Zhao et al., 2018), cold, aluminum and iron deficiency (Colangelo and Guerinot, 2004; Li et al., 2016; Zhang et al., 2015). In addition, the bHLH genes play an important role in regulating multiple signal transduction pathways and impacting secondary metabolite biosynthesis (Zhang et al., 2019). A critical aspect of bHLH function is their ability to form transcriptional complexes with other transcription factors, enabling precise regulation of gene expression. For instance, bHLH transcription factors often interact with MYB proteins, forming MYB-bHLH-WD40 (MBW) complexes that control the regulation of anthocyanin biosynthesis in various plants (Ramsay and Glover, 2005; Zhou et al., 2022). In apples, the interaction between MdbHLH3 and MdMYB10 enhances anthocyanin production and contributes to fruit coloration (Espley et al., 2007). Similarly, in *Arabidopsis*, MYC2 interacts with other bHLH transcription factors, such as MYC3 and MYC4, to form transcriptional complexes that regulate the JA-mediated response to environmental stresses and defense against herbivores (Fernández-Calvo et al., 2011). Furthermore, bHLH proteins are involved in regulatory networks that coordinate with other transcription factor families, such as APETALA2/Ethylene Response Factors (AP2/ERF), WRKY, and NAC transcription factors. These interactions can modulate diverse biological processes, such as root development, leaf senescence, and responses to environmental stressors. For example, the bHLH transcription factor ILR3 forms complexes with other factors to control iron homeostasis in response to iron deficiency (Li et al., 2016; Yang et al., 2024).

Sea buckthorn (Hippophae rhamnoides) is a hardy, fast-growing, deciduous, spiny shrub, naturally distributed in Asia and Europe (Zhang et al., 2018, 2019, 2017). Sea buckthorn fruit and leaves have high levels of vitamins C, E and A, organic acids, amino acids, fatty acids, carotenoids and flavonoids (Pop et al., 2014; Tiitinen et al., 2005, 2006). Due to its special nutritional and healthy value, sea buckthorn was considered as source of valuable nutrients for nutraceuticals and cosmeceuticals. Completing the whole genome sequencing of sea buckthorn makes it possible to investigate the genomic organization, gene structure, evolution and function of the bHLH gene family at the genome-wide scale^31^. In this study, 144 HrbHLH genes were identified and investigated for their structures and functions. In addition, differential gene expression was also carried out to identify the expression patterns during different fruit developmental stages to obtain some candidate HrbHLH genes involving secondary metabolite biosynthesis in sea buckthorn.

## Materials and methods

### Plant materials and growth conditions

The *H. rhamnoides* L. subsp. *Mongolica Rousi* was used in this study. All these sea buckthorns were planted in the Desert Forest Experimental Center in Inner Mongolia, China. The present study’s use of plants/plant parts complies with institutional, national, and international guidelines. Desert Forest Experimental Center in Inner Mongolia is affiliated with the Chinese Academy of Forestry. Professor Jianguo Zhang has officially appraised the materials, and the voucher sample is now formally stored in the Desert Forest Experimental Center in Inner Mongolia. Healthy fresh sea buckthorn fruits were harvested at 30, 42, 49, 56 and 63 days post-anthesis (S1, S2, S3, S4 and S5), and quickly placed in the liquid nitrogen and stored at -80°C until the next use.

### Identification and characterization of the sea buckthorn bHLH gene family

The genome-wide protein sequences and GFF file of sea buckthorn were downloaded from the H. rhamnoides Information Archive database (http://hipp.shengxin.ren/). The Hidden Markov Model (HMM) profile of the bHLH domain (PF00010) was downloaded from the Pfam database (Finn et al., 2016), and was used as a query to scan the proteome file via HMMER software (version 3.1) with a default E-value (Kitony et al., 2021; Mistry et al., 2013). The protein sequences for bHLH genes shown in those HMMER results were obtained from the proteome file using TBTools(https://www.tbtools.com/). Redundant sequences were removed with online ElimDupes software, and a few sequences with obvious errors were removed manually. MapInspect software was used to map the sea buckthorn bHLH genes on different chromosomes, and annotation data in the GFF file were exhibited by the online tool WEGO2.0 (Ye et al., 2018). The lengths, masses, isoelectric point (PI)-values, and charge at pH7.0 for these bHLH protein sequences were determined with DNAstar software, and length distributions and functional annotations were analyzed with Blast2GO software (Conesa et al., 2005).

### Multiple sequence alignment and phylogenetic analysis

144 predicted HrbHLH proteins, with amino acids spanning the bHLH core domain, were subjected to a multiple sequence alignment using ClustalX 2.0 with the default parameters. The further multiple sequence alignment was performed using ClustalW 2.0 (Larkin et al., 2007). The phylogenetic tree representing HrbHLH proteins was generated using MEGA 7.0 software and the Maximum likelihood method, with the following settings: mode, “p-distance”; gap setting, “Complete Deletion”; and bootstrap test replicate, ‘1,000’ (Kumar et al., 2016).

### Gene structure and conserved motifs analyses of HrbHLH genes

Information about the gene structure (intron-exon) of each putative bHLH gene was obtained from the GFF file, downloaded from the GDR database. The schematic structures of these genes were drawn with the online Gene Structure Display Server (GSDS2.0) (Hu et al., 2015). Local MEME software was used to identify conserved motifs in the protein sequences using the following parameters: -protein, -oc m12, -mod zoops, -nmotifs 12, -minw 6, and -maxw 70 (Bailey et al., 2009). The results from these analyses of gene structure and conserved motifs were arranged according to the order shown on the phylogenetic tree.

### 3D protein homology modeling and protein properties

First, BLASTP search with the default parameters was performed in the Protein Data Bank (PDB) with all bHLH proteins to identify the best template with a similar sequence and known three-dimensional structure. Using ‘intensive’ mode in Protein Homology/Analogy Recognition Engine (Phyre2) (Kelley et al., 2015), the data was analyzed for the prediction of protein structure of sea buckthorn bHLHs. The theoretical isoelectric point (PI) and protein statistics were analyzed using ExPASy and Sequence Manipulation Suite, respectively.

### Expression analysis of HrbHLH genes in sea buckthorn

To evaluate the sea buckthorn bHLH gene expression patterns, we downloaded the Illumina RNA-seq data of sea buckthorn different tissues from *H. rhamnoides* Information Archive database (http://hipp.shengxin.ren/), including root nodule, root, leaf, steam and fruit tissues. Each sample has three biological repeats. First, all these raw data were spitted and converted to fastq format file by the NCBI SRA Toolkit’s fastq-dump command. The quality of fastq files was evaluated with FASTQC (http://www.bioinformatics.babraham.ac.uk/projects/fastqc/). Also, low-quality reads were trimmed with local perl script. After the final quality check, RNA-seq sequence datasets were aligned to the whole sea buckthorn genome using TopHat2 (Kim et al., 2013). Normalization of the gene expression values was done by the fragments per kilobase of exon model per million mapped reads (FPKM) algorithms (Shi et al., 2020). The heatmaps of hierarchical clustering were visualized with OmicShare tools(http://www.omicshare.com/tools).

### RNA extraction and real-time quantitative qRT-PCR analysis

Plant materials were harvested, frozen in liquid nitrogen, and ground under RNase-free conditions. The RNA was extracted with TRizol reagent (Invitrogen), following the manufacturer’s instructions, and then treated with DNase I at 37°C for 30 min. The RNA (1 µg) was then reverse-transcribed using a PrimeScript First-strand cDNA Synthesis Kit (Takara, Dalian, China) according to the manufacturer’s instructions. A 10-µL aliquot of cDNA was diluted to 100 µL with water, and 2 µL of that diluted cDNA was used for the analyses. For real-time quantitative qRT-PCR, gene specific primers for our selected HrbHLHs were designed and synthesized by Sangon Biotech (Shanghai, China) Company (product size 110-130 bp; Tm 59-61°C; details are shown in **Supplementary Table S1**), and Hr18S was used as an internal control. All reactions were performed on an Icycler iQ5 system (Bio-Rad), using the SYBR Green Supermix Kit (Bio-Rad) according to the manufacturer’s instruction. Expression levels of these genes were calculated as 2^ΔCT^ values. Besides, relative expression levels for each gene along the time series were also calculated as 2^ΔΔCT^ values. At least three biological replicates were used for the fluorescence-quantitative PCR reactions, with each biological repeat having at least three technical replicates. Each biological repeat contains at least 6 plantlets for mixing.

## Results

### Identification, chromosomal locations, and functional annotation of sea buckthorn bHLH genes

**Figure 1:**
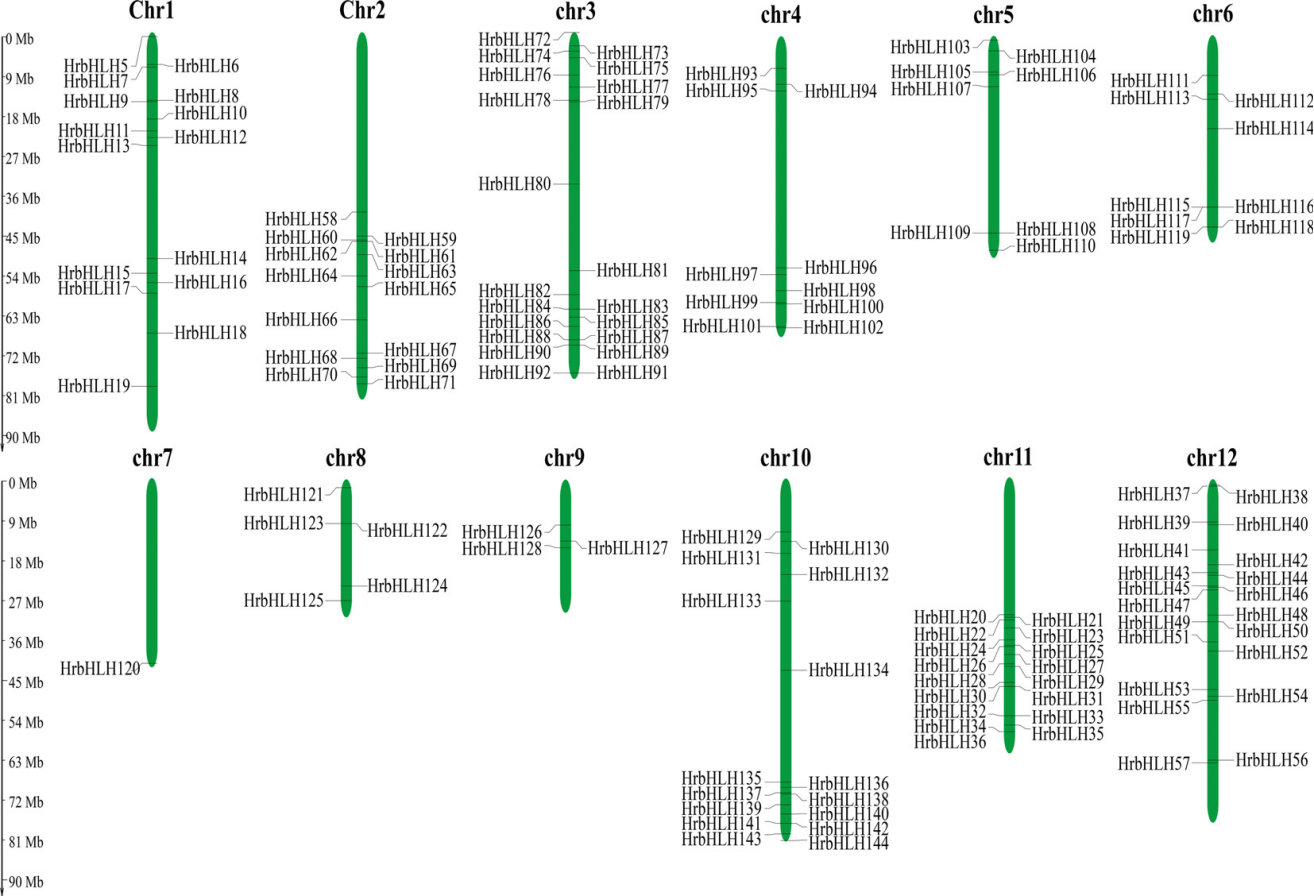
Distribution of 144 HrbHLH genes onto twelve sea buckthorn chromosomes. The position of each HrbHLH is noted on the right or left side of each chromosome (chr). Scale bar, 9 Mb.

To identify HrbHLH genes at the genome scale, we search the sea buckthorn proteome using the HMM files. A total of 180 putative HrbHLH protein sequences were obtained. After the existence of the conserved bHLH domain was confirmed by SMART and CD-Search. After removing redundant sequences, we finally identified 144 bHLH proteins in the sea buckthorn genome. Based on their chromosomal locations, these sea buckthorn bHLH genes were named from HrbHLH1 to HrbHLH144 (**Figure 1**). Sequence analysis revealed that these HrbHLH proteins vary widely in length and have an average of 326.5 aa. The most length of HrbHLH proteins was HrbHLH76 (758 aa), and the least length of HrbHLH proteins was HrbHLH129 (92 aa). Their predicted molecular weights ranged from 10.23 kDa to 81.93 kDa, with an average of 36.47 kDa. Their predicted pI values ranged from 4.50 to 10.61. Gene IDs, genomic positions, and annotation information were also summarized for these HrbHLH proteins (**Supplementary Table S1**).

According to the genomic position of putative HrbHLH genes, we showed that the HrbHLH genes were found across all 12 chromosomes, ranging from 1 to 21 per chromosome (**Figure 1**). However, the other four bHLHs (HrbHLH1-HrbHLH4) were localized to unassembled genomic contigs and cannot be mapped to any particular chromosome according to what we currently know about this genome (**Figure 1**). Chromosome 3 and 12 has the most HrbHLHs (21 total), followed by chr11 (17 genes) and chr10 (16 genes). The sea buckthorn genome database provided GO and KEGG annotation information about these HrbHLH proteins. Among these HrbHLH, three HrbHLH genes (*HrbHLH7*, *HrbHLH133*, *HrbHLH39*) were related to organ development. In addition, two HrbHLH genes (*HrbHLH120* and *HrbHLH107*) were enriched into the plant hormone signal transduction pathway.

### Phylogenetic analysis and prediction of conserved motifs

**Figure 2:**
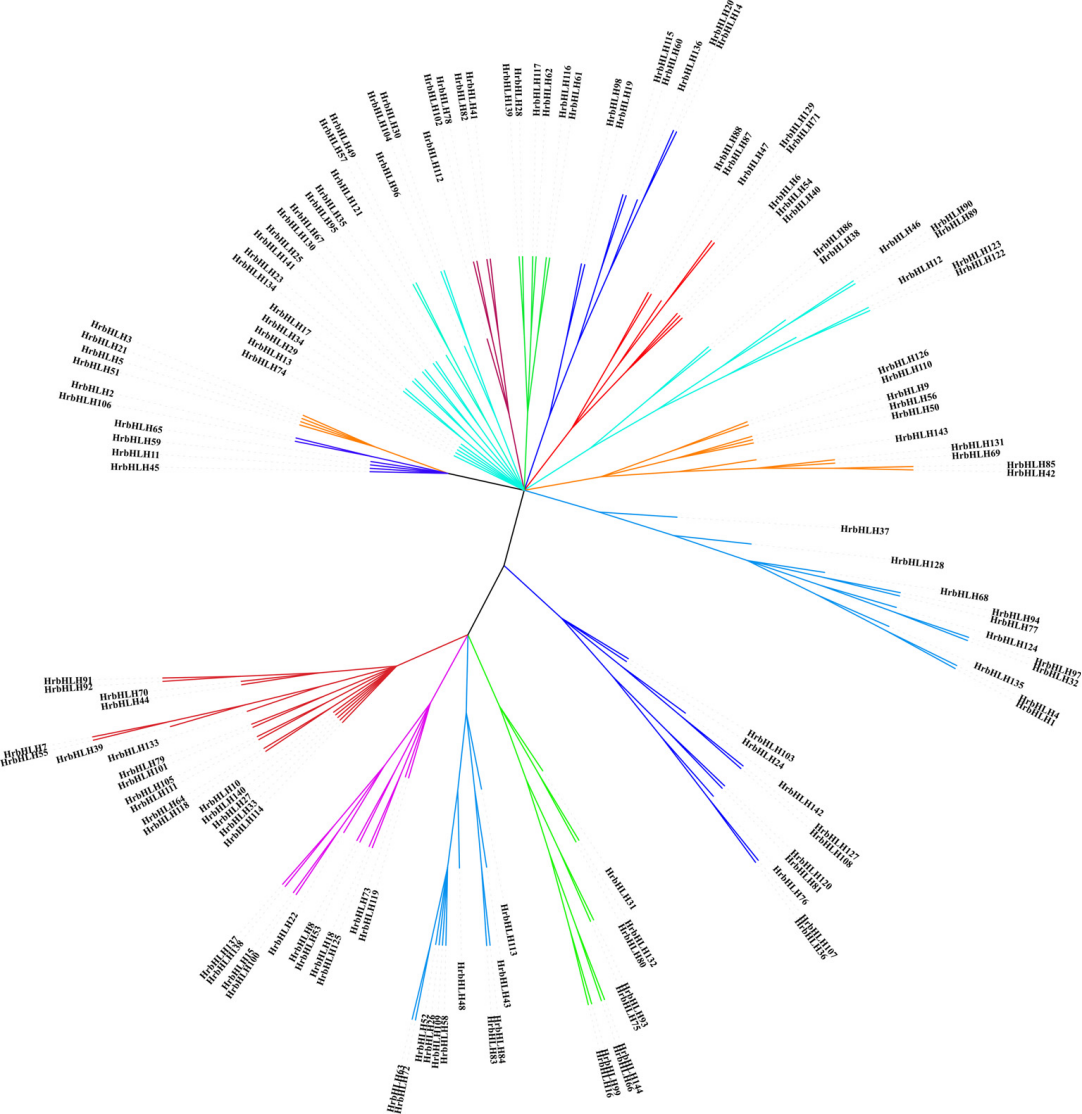
Phylogenetic tree of sea buckthorn, *Arabidopsis* and rice bHLH proteins. The Neighbor-joining phylogenetic tree was constructed with MEGA7 software.

**Figure 3:**
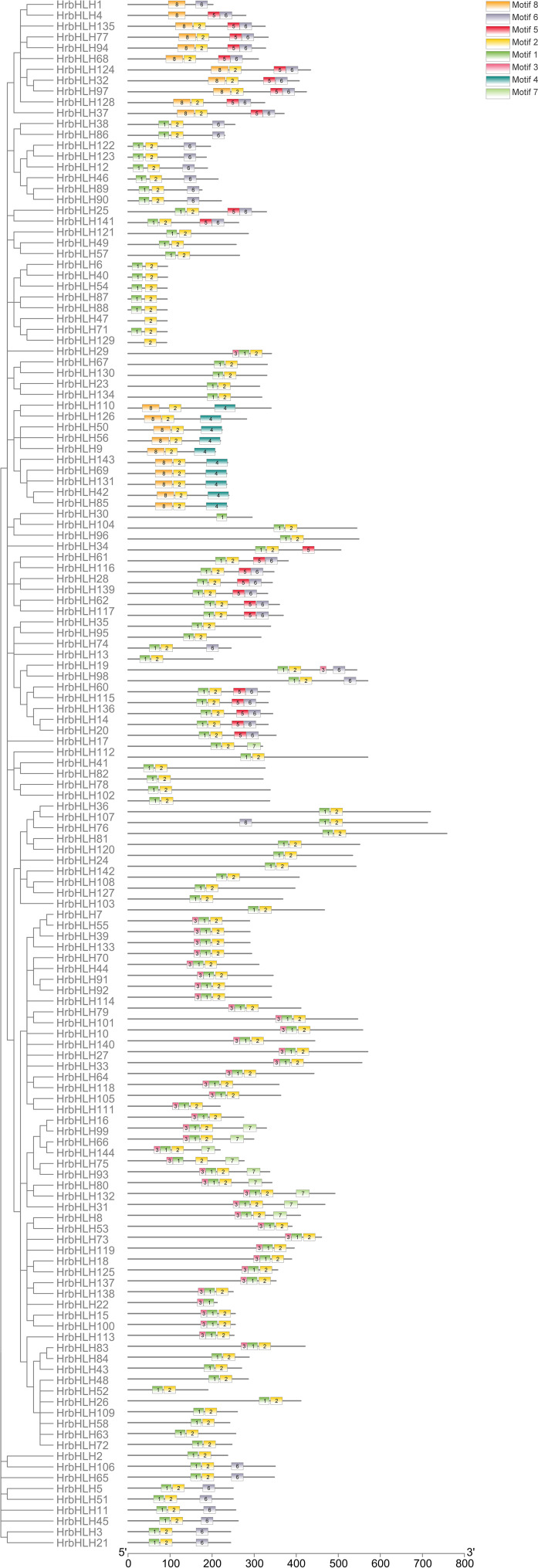
Conserved motifs of HrbHLH proteins. Arrangements of conserved motifs in the HrbHLH proteins. Different colored boxes represented seven different motifs. All HrbHLH proteins contained Motif 1 and Motif 2, while HrbHLH in groups G6 and G7 didn’t have Motif 1. Also, all HrbHLHs in groups G6 and G7 contained Motif 8.

The number of plant bHLH protein classifications ranged from 15 to 32 in different species. Using NJ method, we conducted a phylogenetic analysis based on full-length protein sequences to evaluate the evolutionary relationships of *Arabidopsis* and rice HrbHLH proteins. All HrbHLH proteins were divided into 25 main groups (G1-G25) (**Figure 2**). The average size of the groups has approximately 9 members, ranging from 1 to 12. Group 20 only has the one member (HrbHLH74). The conserved DNA binding domain sequences of sea buckthorn bHLH family are identified and displayed in **Figure 3**, which contained a lot of conserved amino acid residues. Five residues (His-11, Glu-15, Arg-16, Arg-18, and Arg-19) are highly conserved in the basic regions. Additionally, the first helix region contained five conserved residues (Ile-25, Asn-26, Arg-28, Leu-32, Leu-35, Val-36, and Pro-37), or in the second helix region (Lys-44, Ala-45, Ser-46, Leu-48, Ala-51, Ile-52, Tyr-54, Lys-56, and Leu-58).

We identified eight putative conserved protein motifs in HrbHLH family proteins using MEME online software (Motifs 1-8, **Figure 3**). All HrbHLH family proteins contained Motif 1 and Motif 2, while HrbHLH in groups G6 and G7 didn’t have Motif 1. Also, all HrbHLHs in groups G6 and G7 contained Motif 8. In each group, the components of the conserved motifs for most of the proteins were similar. For example, Motifs 1, 2, and 6 were identified in all eleven members of group G2, and Motifs 1, 2, and 3 were identified in all 12 members of group G1, and Motifs 4 were involved in 11 members of group G2. The evolutionary relationships among these HrbHLH proteins were also determined by analyzing their conserved motifs. These composition patterns tended to be consistent with the results from our phylogenetic tree, being nearly identical among genes within the same group but varying greatly between groups.

### Analysis of gene structures

The distribution pattern of exon and intron of a gene family can also provide important evidence to confirm phylogenetic relationships. We performed gene structure analysis of the 144 HrbHLHs to gain information about exon-intron organization (**Supplementary Figure S1**). Among these 144 sea buckthorn genes, a total of seven bHLH genes (*HrbHLH26*, *HrbHLH48*, *HrbHLH52*, *HrbHLH58*, *HrbHLH63*, *HrbHLH67*, *HrbHLH109*) without intron were found, which accounts for 5% of total HrbHLH genes. All these intron-less genes were clustered into group G3. According to an exon-intron organization of bHLH genes in sea buckthorn, phylogenetically related proteins exhibited a closely related gene structure intron number or exon length.

### 3D homology modeling of HrbHLH proteins

**Figure 4:**
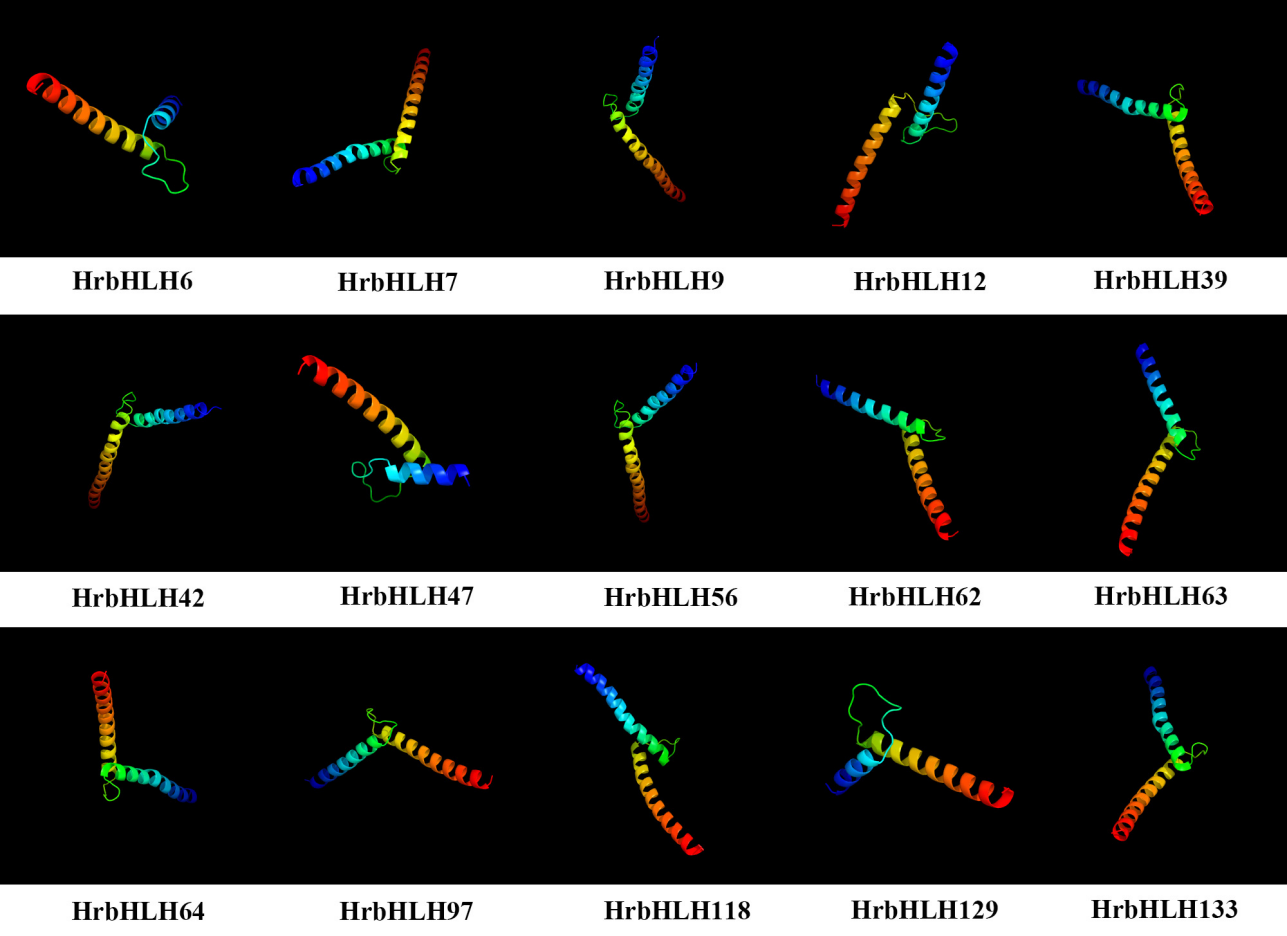
Predicted 3D structures of bHLH proteins in sea buckthorn. The structure of 15 HrbHLH proteins with >90% confidence level is shown.

The 3D protein modelling of selected 15 bHLH proteins is predicted at >90% confidence, and the percentage residue varied from 80 to 100 (**Figure 4**). These bHLH genes have been selected based on their expression patterns in the plant samples studied. All 15 HrbHLH proteins were predominantly constituted of α helices and β sheets. Thus, protein structures provided a preliminary basis for understanding the molecular function of HrbHLH proteins.

### Expression patterns of HrbHLH genes in different tissues

To further understand the function of sea buckthorn bHLH proteins, the expression patterns of sea buckthorn bHLH genes among 21 samples, including five tissues, were analyzed, according to the normalized RPKM data from RNA-seq. **Supplementary Table S2** shows the expression profiles of all bHLH genes in the 21 sea buckthorn samples. Among the 144 HrbHLH genes, 122 HrbHLH mRNAs had an RPKM value greater than 1 in at least one of the 21 samples, while the remaining 22 HrbHLH genes were expressed at very low levels in all 21 samples. Among these HrbHLH genes, *HrbHLH47*, *HrbHLH74*, *HrbHLH90*, *HrbHLH131* were the highest expression level in root nodule, root, leaf, stem and fruit tissues, respectively.

**Figure 5:**
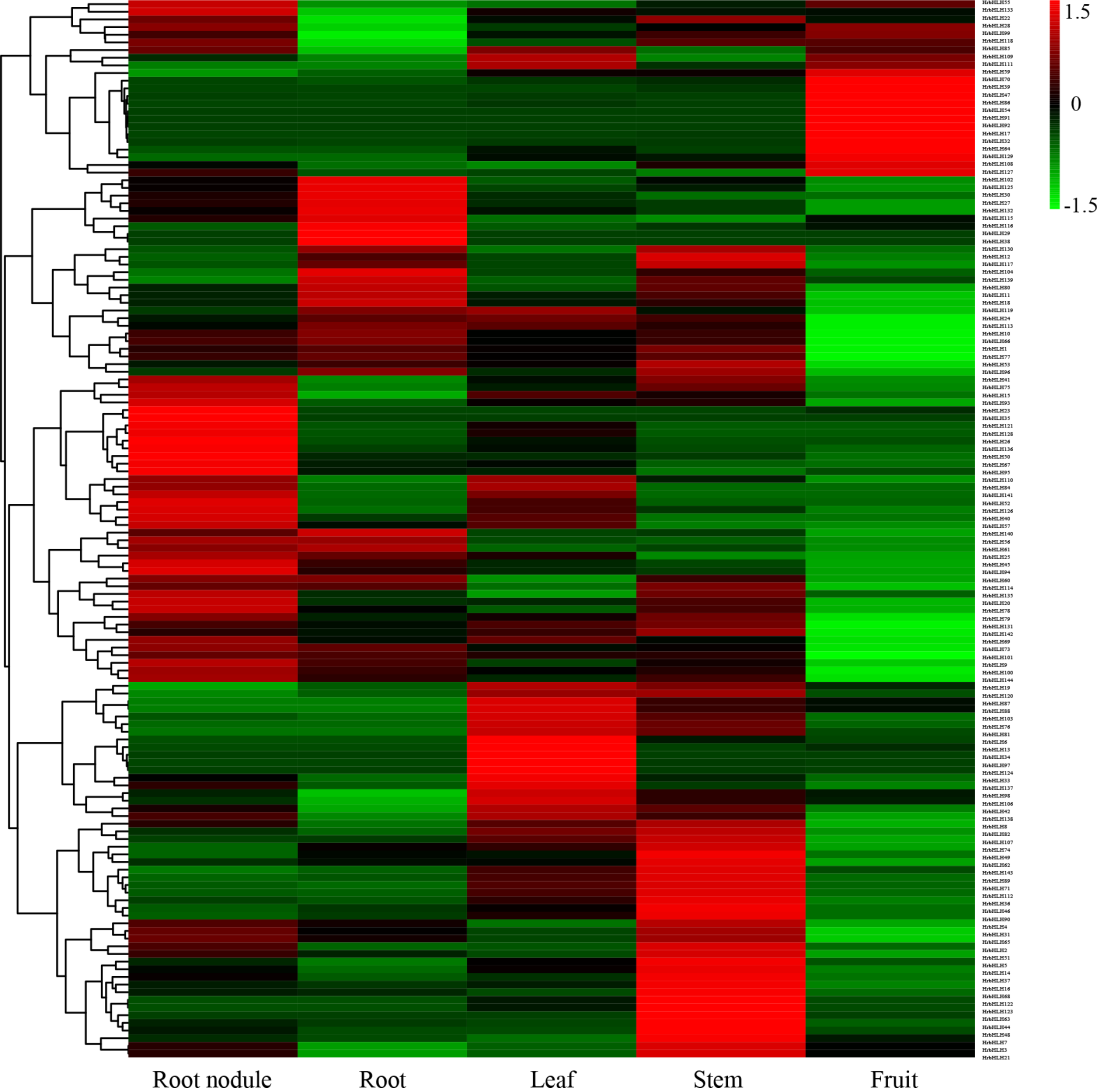
Expression heatmap of HrbHLH genes in root nodule, root, leaf, stem and fruit. Each sample have three repeats. The bHLH gene name were on the right of the figure. The color scale is shown at the top. Higher expression levels are shown in red. Lower expression levels are shown in green.

**Figure 6:**
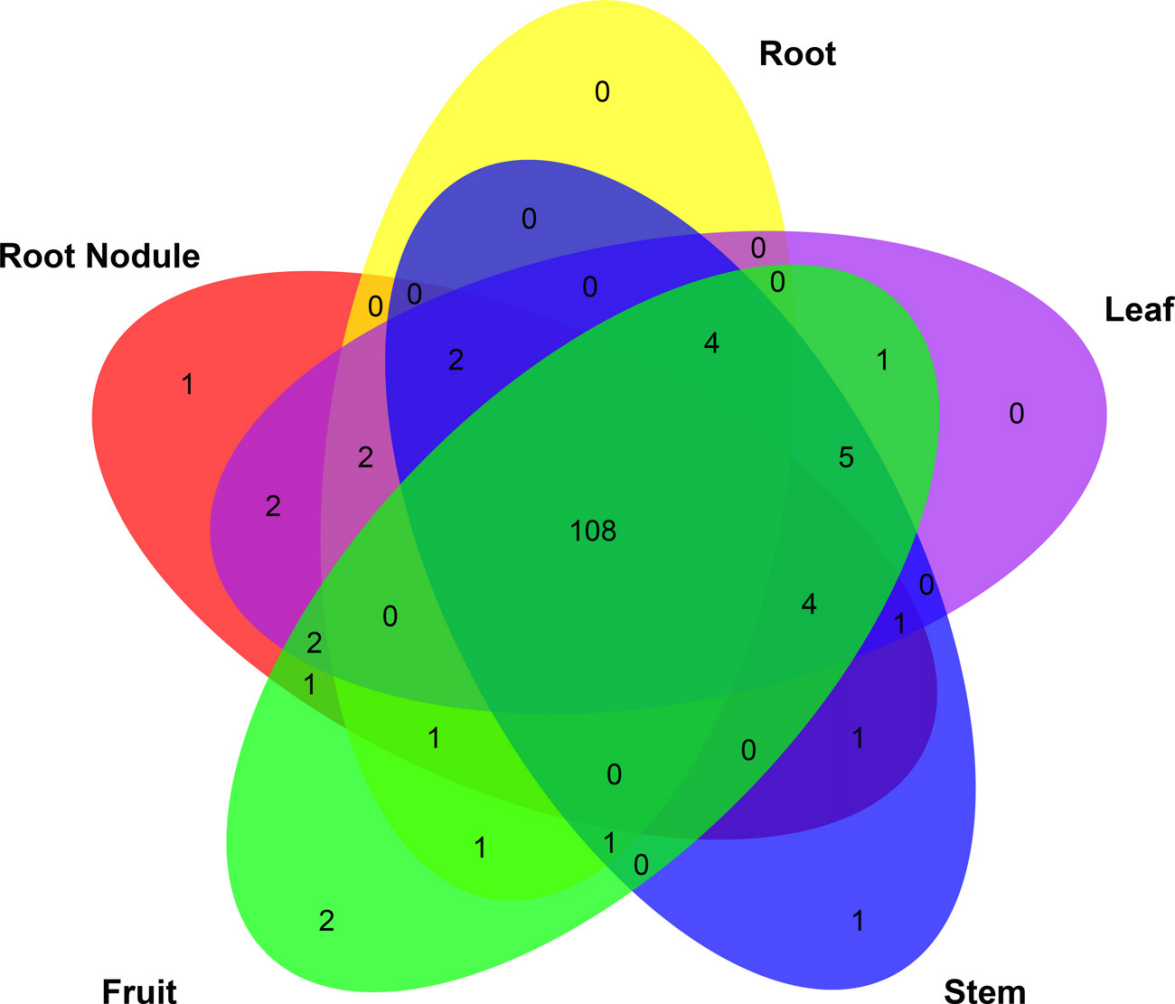
Venn of expressed HrbHLH gene in different tissues. Among 144 HrbHLH genes, 108 were expressed in all these five tissues.

In particular, *HrbHLH9*, *HrbHLH42*, *HrbHLH69*, *HrbHLH74*, *HrbHLH75*, *HrbHLH85*, *HrbHLH90* and *HrbHLH131* were constitutively produced at a relatively high level in all 21 samples, suggesting that these eight bHLH genes perform a variety of functions in different tissues (**Figure 5**). Furthermore, four HrbHLH genes showed preferential tissue-specific expression, including one gene in root nodule (*HrbHLH35*), two genes in fruits (*HrbHLH43* and *HrbHLH58*), one gene in stem (*HrbHLH63*) (**Figure 6**). The specific accumulation of these bHLH genes in a particular tissue suggests they may play conserved regulatory roles in discrete cells, organs, or conditions.

### Expression profiling for HrbHLH genes during sea buckthorn fruit development process

**Figure 7:**
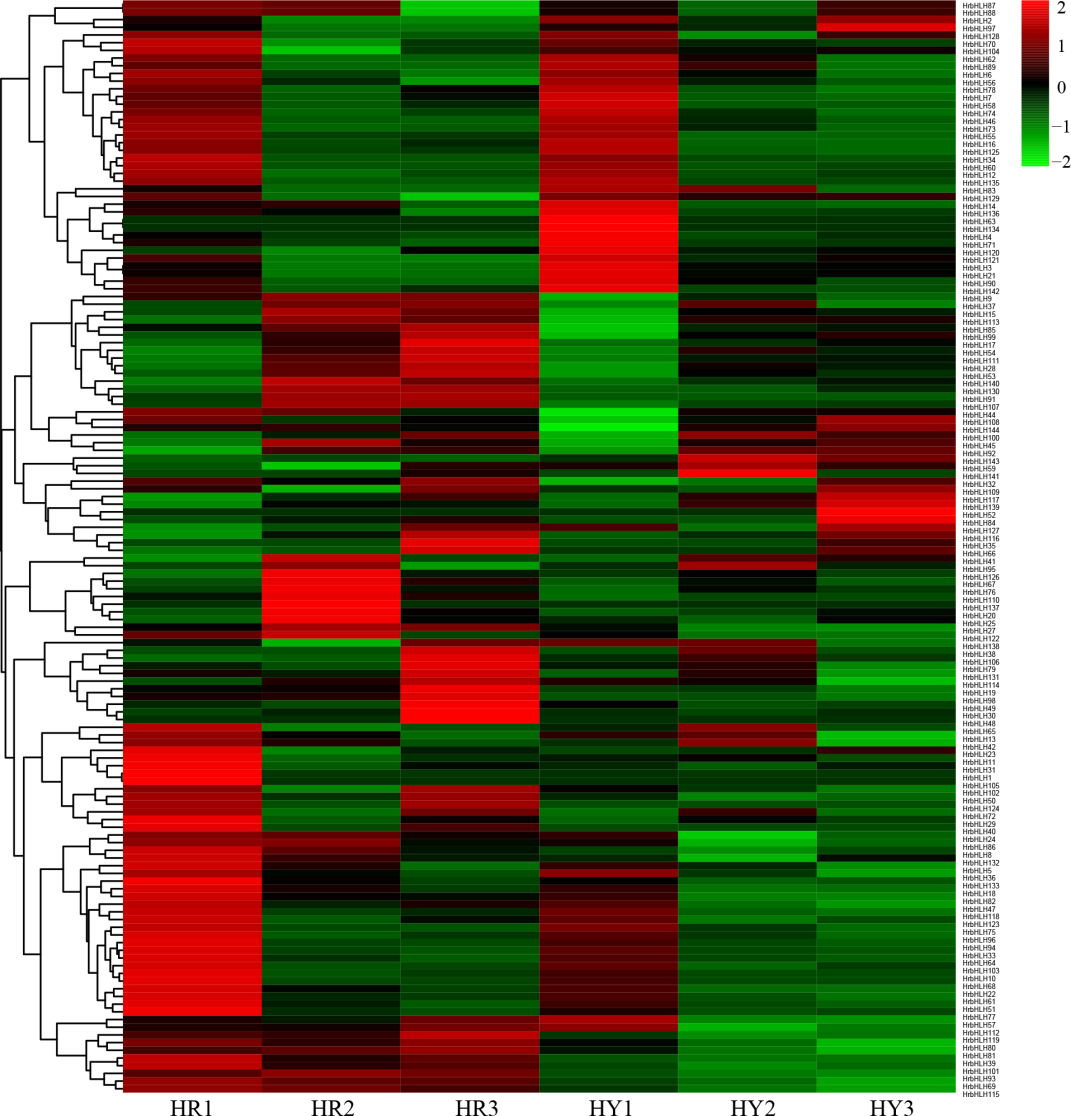
Expression heatmap of HrbHLH genes in different development stages of sea buckthorn fruit. Each sample have three repeats. The bHLH gene name were on the right of the figure. The color scale is shown at the top. Higher expression levels are shown in red. Lower expression levels are shown in green.

To analyze the expression pattern of HrbHLH genes among different development stages, we used the Fragments Per Kilobase per Million (FPKM) normalized data from RNA-seq. The additional file showed the expression profiles of HrbHLH genes in sea buckthorn fruit in different development stages. Among 144 HrbHLHs genes, 131 were expressed at least in one fruit development stage, while the rest HrbHLHs genes were not expressed in sea buckthorn fruit. Based on gene expression data, we identified eleven HrbHLH genes (*HrbHLH27*, *HrbHLH54*, HrbHLH*70*, *HrbHLH79*, *HrbHLH91*, *HrbHLH92*, *HrbHLH100*, *HrbHLH104*, *HrbHLH111*, *HrbHLH127*, *HrbHLH132*) which showed a gradually decreased expression with fruit development and ripening, suggesting that they may function in fruit ripening. Among them, HrbHLH54, HrbHLH91 and HrbHLH92 exhibited a high expression in sea buckthorn ripe fruit, and the rest bHLH gene has low expression level in sea buckthorn fruit (**Figure 7**).

**Figure 8:**
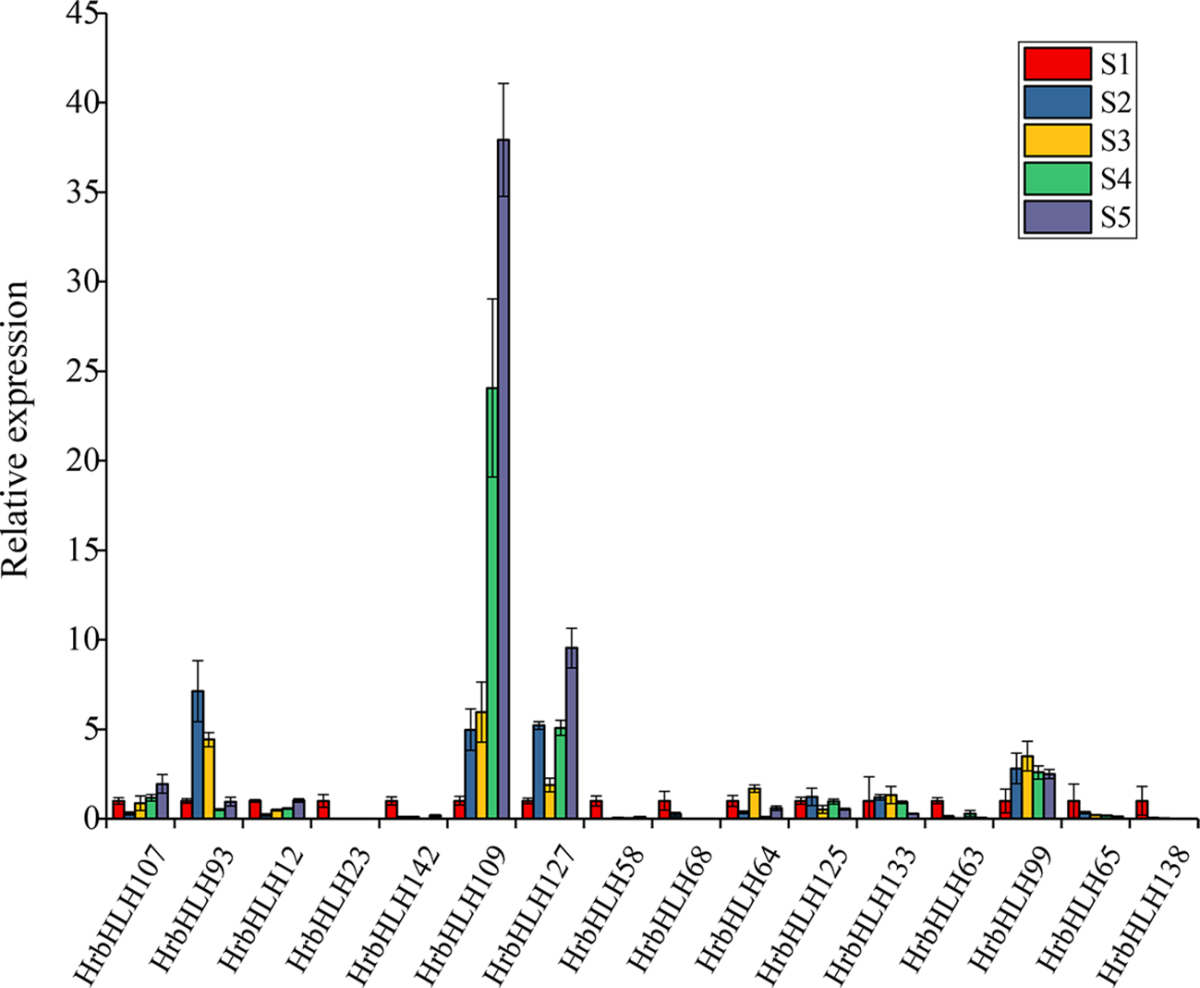
Expression patterns of the HrbHLH candidate genes in different development stages of sea buckthorn fruit by qRT-PCR. S1-S5: five development stages of sea buckthorn fruit.

Furthermore, we identified 54 HrbHLH genes which showed a gradually increased expression with fruit development and ripening. Compared with down-regulated bHLH genes, these up-regulated bHLH genes have lower expression levels. And the expression level of 16 HrbHLH genes was confirmed with qRT-PCR (**Figure 8**). Therefore, further characterization of the eleven HrbHLHs with decreased expression levels is highly important and will provide new insight to understand the molecular mechanism of fruit development and ripening.

## Discussion

Sea buckthorn is an important economic species of the Elaeagnus family (Tiitinen et al., 2005). The sea buckthorn fruit is rich in nutrients, which can provide essential amino acids and have antioxidant activity (Tiitinen et al., 2006). With the sea buckthorn genome sequencing finish, we can identify and characterize transcription factor gene family at the whole genome level (Chu and Wei, 2020). Studying the transcription factor family of sea buckthorn holds the promise for breeding excellent cultivars and improving fruit quality. However, no detailed studies have been done on the bHLH family in sea buckthorn.

As the second largest transcription factor family in eukaryotic kingdoms, bHLH superfamily plays important role in many biochemical and physiological processes (Heim et al., 2003). This superfamily is divided into 20-25 subfamilies in important plants according to conserved domains and their evolutionary relationships. Many studies have been reported on the bHLH superfamily-related genes in many species, including cucumber, *Arabidopsis*, apple, tomato and *Carthamus tinctorius*. In addition, their functional and structural characterizations have also been described. All these results indicated that bHLH proteins are involved in different biochemical and physiological processes in the plant kingdom (Reyes et al., 2021). In this study, we identified 144 HrbHLH genes in sea buckthorn genome, less than bHLH genes in tomato and apple genome (Xu et al., 2018; Zhu et al., 2017). Based on phylogenetic analysis, predicted conserved protein motifs and intron-exon organizations, all identified HrbHLHs were divided into 15 groups, different from other species (Carretero-Paulet et al., 2010; Heim et al., 2003). These results indicate that sea buckthorn bHLHs have different classification models with other plants. Furthermore, our intron-exon gene structure and conserved protein motifs result strongly supported our classifications of the HrbHLHs.

Most of these HrbHLH genes showed tissue-specific expression patterns, which suggested their roles in regulating the corresponding organogenesis. Thus, the expression profile analysis provided a potential proof of function for this kind of proteins (for example, HrbHLH35 was predominantly expressed in root nodules, indicating that it may participate in nitrogen fixation or root development). High expression levels of HrbHLH43 and HrbHLH58 were detected in fruits, which inferred their potentials to be involved in fruit-specific regulatory pathways. The expression of HrbHLH63 was predominantly concentrated in the stems, suggesting its potential role in stem development and/or integrity. These included genes HrbHLH9, HrbHLH42, HrbHLH69, HrbHLH74, HrbHLH75, HrbHLH85, HrBhlh90 and HRBhlh131 that showed constitutive expression across all 21 tissue samples investigated suggesting they might be involved in maintaining essential biological functions common to several tissues (Yao et al., 2017). These genes are strong candidates for further study to elucidate their roles in essential plant growth and development pathways. The specific accumulation of these bHLH genes in a particular tissue suggests they may play conserved regulatory roles in discrete cells, organs, or conditions (Kitony et al., 2021; Xu et al., 2018).

The bHLH transcription factor is a critical regulator in many biological processes, such as cell differentiation, organ development, and environmental signaling (Liu et al., 2020). Several bHLH genes have been identified to play important roles in fruit development in species including tomato, pyrus, apple, and almond during recent years. This study serves to underscore the necessary role for bHLH transcription factors controlling implementation of developmental processes, from growth in cell size to ripening Seabuckthorn. Among them, HrbHLH47 and HrbHLH90 were identified in sea buckthorn as crucial transcripts regulating fruit enlargement. Together, they show very high expression during early fruit development, indicating their role in processes associated with cell expansion leading to an increase in fruit size. HrbHLH47 has a high expression level across the whole fruit development process. HrbHLH47 is the homolog of AtPRE5, a key factor regulating cell elongation and plant development. The homologous comparison and protein interaction prediction also indicated that it might be involved in fruit enlargement (Groszmann et al., 2011; Zhu et al., 2017). The expression HrbHLH90 was significantly higher at the early stage of fruit development. This result also indicated that it might be involved in fruit enlargement. In addition, the expression of HrbHLH91 and HrbHLH92 were significantly higher at the ripen stage of fruit development.

Across other species, bHLH transcription factors have been shown to regulate various aspects of fruit development and ripening, often through hormonal and stress-responsive pathways. Insights from these systems provide valuable clues for understanding the functions of bHLH genes in sea buckthorn. For instance in tomato, bHLH genes like SlPRE2 and SlPRE3 have been linked to early fruit development, while others, like SlbHLH59, control ripening through ethylene-mediated pathways (Groszmann et al., 2011). The identification of homologous functions for HrbHLH47 and HrbHLH90 in sea buckthorn suggests that similar mechanisms may be conserved across species, regulating both the physical enlargement of fruits and the subsequent ripening process. In apple, bHLH transcription factors have been implicated in regulating anthocyanin biosynthesis and the development of fruit coloration. Genes such as MdMYC2 are involved in modulating the production of secondary metabolites that contribute to fruit quality (Zhang et al., 2019). (The high expression of HrbHLH91 and HrbHLH92 during sea buckthorn ripening suggests that these genes may play similar roles in enhancing fruit pigmentation and flavor, providing a potential target for improving fruit aesthetics and nutritional content in breeding programs. Similarly, in almond and pyrus, bHLH genes have been shown to regulate not only fruit size but also kernel or seed development (Li et al., 2019). Although sea buckthorn does not produce large seeds, the comparative analysis of bHLH genes involved in nutrient allocation could provide insights into how HrbHLH genes contribute to the overall fruit nutritional profile.

### Implications for future research

The identification of these key HrbHLH genes provides some of the best permitive skeletal analysis as well. All aspects concerning the development of HrbHLH47, HrbHLH90, HrbHLH91, and HrbHLH92 should be investigated in detail, as they are promising targets towards sea buckthorn breeding strategies aimed at enhancing fruit size, quality, and nutritional value, particularly bioactive phytochemicals. Functional studies in sea buckthorn or other model systems such as *Arabidopsis* using gene knockout or overexpression approaches in particular could help to elucidate how these HrbHLH genes control these specific developmental events. Furthermore, the manipulation of these genes may enable the control of plant growth and development under various environmental conditions through alteration of hormone pathways. Our study reports the first comprehensive genome-wide analysis of the bHLH gene family in sea buckthorn. It helps to develop further HrbHLH gene function investigations, which will facilitate the development of sea buckthorn cultivars in molecular breeding programs and genetic engineering.

## Data Availability

The original contributions presented in the study are publicly available. This data can be found on the NCBI database with the below accession numbers: SRR27862265; SRR27862264; SRR27862263; SRR27862262; SRR27862261; SRR27862260; SRR27862259; SRR27862258; SRR27862257.
