# Supplementary figures and images for: Genome expression analysis of basic helix-loop-helix transcription factors in Sea buckthorn (Hippophae rhamnoides L.)
Source: Front Plant Sci. 2024 Nov 20;15:1487960. doi: 10.3389/fpls.2024.1487960 (PMC11614652; doi:10.3389/fpls.2024.1487960)

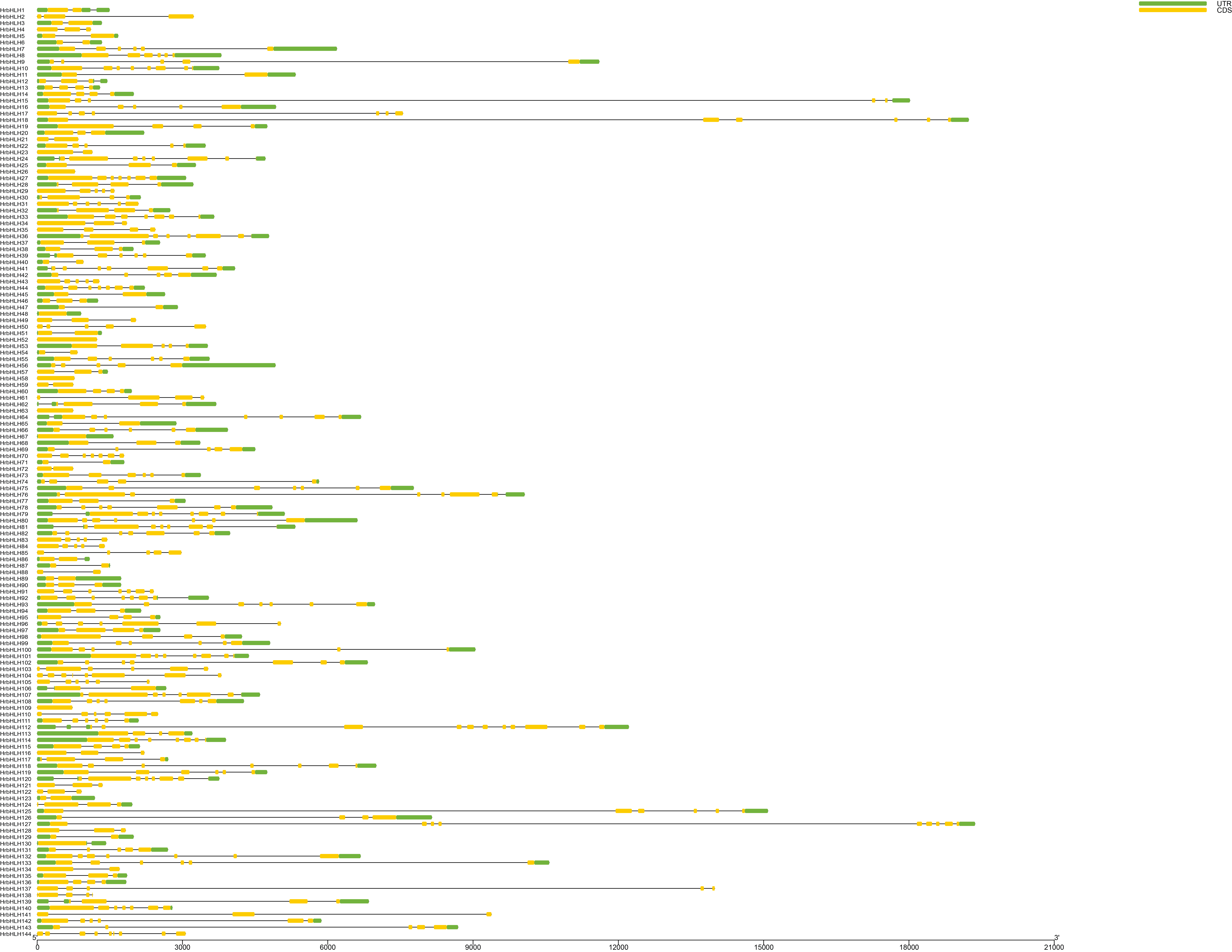

Supplement: Supplementary file 1 [file Image1.tif]
